# Supplementary material for: Curated and harmonised transcriptomics datasets of interstitial lung diseases
Source: Data Brief. 2025 Oct 14;63:112139. doi: 10.1016/j.dib.2025.112139 (PMC12581653; doi:10.1016/j.dib.2025.112139)
Supplement: Supplementary file 1 [file mmc1.zip › Supplementary_material/RNA-seq/fastQC reports/GSE184316/SRR15926718_fastqc.html]

SRR15926718.fastq.gz FastQC Report 

FastQC Report

Mon 5 Feb 2024  
SRR15926718.fastq.gz

## Summary

- Basic Statistics
- Per base sequence quality
- Per sequence quality scores
- Per base sequence content
- Per sequence GC content
- Per base N content
- Sequence Length Distribution
- Sequence Duplication Levels
- Overrepresented sequences
- Adapter Content

## Basic Statistics

| Measure | Value |
| --- | --- |
| Filename | SRR15926718.fastq.gz |
| File type | Conventional base calls |
| Encoding | Sanger / Illumina 1.9 |
| Total Sequences | 14460357 |
| Sequences flagged as poor quality | 0 |
| Sequence length | 25-379 |
| %GC | 50 |

## Per base sequence quality

## Per sequence quality scores

## Per base sequence content

## Per sequence GC content

## Per base N content

## Sequence Length Distribution

## Sequence Duplication Levels

## Overrepresented sequences

| Sequence | Count | Percentage | Possible Source |
| --- | --- | --- | --- |
| CAATCCACTTGATCCCAACTCATCTCTCATTTATTTCGGCTTCTTTTATT | 77144 | 0.533486137306292 | No Hit |
| TTACAAAAGCAGAATAAAAGCGAAAAGAAATGAAAATGTTACACTACATT | 76229 | 0.5271584926983476 | No Hit |
| CAGTGGGGCCACGAGCTGAGTGCGTCCTGTCACTCCACTCCCATGTCCCT | 74205 | 0.513161604516403 | No Hit |
| AGACATCAAATCCTGCAGACAAGGGGAGCCCTCAGTCTGCAGGGCTCCAT | 73893 | 0.5110039814369728 | No Hit |
| CTTCATGATGTAGCAGCAGGTGCCAGGGGCTGGCTTGTAGGCGATCAGCA | 69810 | 0.4827681640225065 | No Hit |
| TACCACTGCCACCTTCTCCATCGGCTCCACTGGCCTCGTGGTGTATGACT | 67919 | 0.46969103183275485 | No Hit |
| GGCATCAAAAGTGATGGACTGCCCATTGCTGGAGAAGACCTTCTCTCCTA | 62537 | 0.432472033712584 | No Hit |
| CACCTCCCCGAAGCTCAGGGAGAGCCCTGTTAGGGCCGCCTCTGGCCCTA | 61082 | 0.4224100414671643 | No Hit |
| TGATATGGTTCCTGGCAAGCCCATGTGTGTTGAGAGCTTCTCAGACTATC | 55520 | 0.38394626080116834 | No Hit |
| TGATGACACCCACCGCAACTGTCTGTCTCATATCACGAACAGCAAAGCGA | 50677 | 0.35045469485988484 | No Hit |
| AGGCTACACACTGAGGACTCTGTTCCTCCCCTTTCCGCCTAGGGGAAAGT | 48081 | 0.33250216436565155 | No Hit |
| TTCAGCCCTGATCAAGACATGAGGGAGGCAGGGGCTCAGCTGAAGAAGCT | 44818 | 0.3099370229932774 | No Hit |
| GGGAAGCGGGGAATGTCTACGTGCGTATGCACGTGGCACTCTCTGCCCGA | 35792 | 0.2475180937787359 | No Hit |
| ACAAGAAGTACATCAAAGATTACATGAAATCAATCAAAGGGAAACTTGAA | 34861 | 0.24107980183338487 | No Hit |
| CTCTCTTTCTGGCCTGGAGGCTATCCAGCGTACTCCAAAGATTCAGGTTT | 33778 | 0.23359036018267046 | No Hit |
| TTTTCCATGAGCTTAATGATGCTTTCTCTGGGCTTTTGGGGGAGGGTGTC | 29118 | 0.20136432316297587 | No Hit |
| GAAACCCAGACACATAGCAATTCAGGAAATTTGACTTTCCATTCTCTGCT | 27563 | 0.19061078505876447 | No Hit |
| GTTCTGCAGCCCCTGTCATAAAAGGTTTTACTCTTTCTGGTCTCTGTTCT | 27154 | 0.18778236249630628 | No Hit |
| GGAAGACAGTGTGGCGATTCCTCAGGGATCTAGAACTAGAAATACCATTG | 25445 | 0.17596384376955562 | No Hit |
| GGCAATGAGCGGTTCCGCTGCCCTGAGGCACTCTTCCAGCCTTCCTTCCT | 23871 | 0.1650789119521738 | No Hit |
| GTCCACGTCACACTTCATGATGGAGTTGAAGGTAGTTTCGTGGATGCCAC | 22020 | 0.15227839810593888 | No Hit |
| CAGCATGTTCCCTCTCCTCATGAGATTGGTGAAGAAAGTATTTGGCAAAG | 21490 | 0.14861320505434272 | No Hit |
| ACGTTTACCTGTCCATGTCTTACTACTTTGACCGCGATGATGTGGCTTTG | 21340 | 0.14757588626615512 | No Hit |
| AGCTGCTAAGTGCTGTGTTGTCGTTCCCCCTGCTTAAAATAAAGTTGTTT | 21012 | 0.14530761584931826 | No Hit |
| CCCTGGCTGCTACAGGAGAATAGCAGACAGGTATAGTTAAGAAACAACTT | 20621 | 0.14260367154144257 | No Hit |
| ATCAAAGAAGAACATGTGATCATCCAGGCCGAGTTCTATCTGAATCCTGA | 18410 | 0.12731359260355743 | No Hit |
| GAAGGACCTCTTCAAGGAGAACTACAAACCACTGCTCAAGGAAATAAAAG | 17291 | 0.11957519444367798 | No Hit |
| CAATGGGGTACTTCAGGGTCAGGATGCCACGCTTGCTCTGGGCCTCGTCG | 16610 | 0.1148657671453063 | No Hit |
| ACCTCTCTCTGGGCTTCTATTTCGACCGCGATGATGTGGCTCTGGAAGGC | 16522 | 0.11425720678956958 | No Hit |
| GGCATCAAAAGTGATGGACTGCCCATTGCTAGAGAAGACCTTCTCTCCTA | 16134 | 0.11157400885745768 | No Hit |
| CGATCAGCACGGGCTCGTACTCTGCCACAAACTGATCACACTGCTTCTGG | 16054 | 0.11102077217042428 | No Hit |
| ATTGATTCTTCCTACCCATGAGCATGGAATGTTCTTCCATTTGTTTGTAT | 15903 | 0.10997653792364877 | No Hit |
| ATAGCAGCATGATTTATAGTCCTTTGGGTATATACCCAGTAATGGGATGG | 15699 | 0.10856578437171363 | No Hit |
| CGAGCCACAGCTCTGCCAGTACCCCAAGACTCAGCACTAGTCTGATGACC | 15417 | 0.10661562504992096 | No Hit |
| TCAGGAGACGCTCGTAGCCCTCGCGCTTCTCCTCGGCCAGTTCGCGGAAG | 15069 | 0.10420904546132576 | No Hit |
| GTCTCAACATCTGTGTTGGGGAGAGTGGAGACAGACTGACGCGAGCAGCC | 14897 | 0.10301958658420399 | No Hit |
| TCAGGAGACGCTCGTAGCCCTCGCGCTTCTCCTCGGCCAATTCGCGGAAG | 14843 | 0.10264615182045643 | No Hit |
| CACAGTCTGCTGGTGGGCAGAGAAGACAGAAACGACATGAGCACAGCAGG | 14526 | 0.10045395144808666 | No Hit |

## Adapter Content

Produced by FastQC (version 0.11.7)
